# Axl signaling is an important mediator of tumor angiogenesis

**DOI:** 10.18632/oncotarget.26882

**Published:** 2019-04-23

**Authors:** Mai Tanaka, Dietmar W. Siemann

**Affiliations:** ^1^Department of Radiation Oncology, College of Medicine, University of Florida, Gainesville, FL, USA

**Keywords:** Axl, angiogenesis, cancer metastasis, BGB324, receptor tyrosine kinase

## Abstract

The growth of primary tumors as well as metastatic neoplastic lesions is strongly dependent on the cancer cells’ ability to initiate their own vascular network. This process, angiogenesis, which involves the proliferation, migration, and invasion of endothelial cells, is critically dependent on a variety of signaling molecules that target specific receptors, most notably tyrosine kinases. One receptor tyrosine kinase associated with poor prognosis, metastasis, and outcome in a variety of tumor types, is Axl. Although the role of Axl in tumor cell migration and invasion are well recognized, little is known about the involvement of Axl signaling in the initiation of angiogenesis. Here, we show that Axl inhibition in tumor cells decreases the secretion of pro-angiogenic factors and impairs functional properties of endothelial cells *in vitro* and *in vivo*. These data indicate that Axl signaling is an important contributor to tumor angiogenesis.

## INTRODUCTION

Angiogenesis, the formation of new blood vessels from pre-existing vasculature, is an important hallmark of tumorigenesis and metastasis [1, 2]. For tumors to grow beyond a size of a few millimeters they must induce new vasculature to meet their growing nutritional needs [3, 4]. In contrast to normal vasculature, the tumor vasculature is highly disorganized, leaky, lacks smooth muscle and pericyte coverage, has intercellular gaps, and abnormal sprouts [5]. These immature vascular networks not only facilitate the dissemination of tumor cells but also induce aberrant physiochemical features within the tumor microenvironment that lead to therapeutic resistance and further promote the aggressive and metastatic behavior of the tumor cells [5–12].

Tumor cells induce angiogenesis by secreting pro-angiogenic factors such as vascular endothelial growth factors, angiopoietins, and releasing proteolytic enzymes that recruit endothelial cells and cleave extracellular matrices [9, 13–16]. Upregulation and activation of receptor tyrosine kinases in tumor cells promote expression of these pro-angiogenic factors and drive the angiogenic process [17, 18]. In addition, these signaling pathways also are essential for tumor progression and metastasis [19–22]. A receptor tyrosine kinase signaling of considerable interest is Axl. Axl belongs in the Tyro-3, Axl and Mer (TAM) subfamily of the receptor tyrosine kinases. Its expression is associated with poor prognosis, metastasis, and outcome in a variety of tumor types including cancers of the breast, prostate, brain, pancreas and ovary [23–29].

Preclinical investigations have demonstrated a role for Axl at multiple steps of the metastatic cascade including cell migration, invasion, proliferation and survival [30–32]. Axl also is expressed on host stromal cells, including endothelial cells [33–35]. Axl inhibition in the human umbilical vein endothelial cells decreased *in vitro* functions associated with angiogenesis, another important step in the metastatic cascade [35].

*In lieu* of the apparent importance of Axl in cancer progression and dissemination, there has been considerable interest in targeting this signaling pathway. Indeed, small molecule inhibitors [36] and monoclonal antibodies [37, 38] targeting Axl have been developed and have gained attention as novel therapeutic agents. A selective Axl inhibitor, BGB324 (R428, bemcentinib), has been shown to inhibit cancer cell metastatic phenotypes of tumor cells *in vitro* and metastatic burden *in vivo* [39, 40]. Currently, this agent is in Phases I/II clinical trials for multiple tumor types (ClinicalTrials.gov Identifier: NCT02922777, NCT03184571, NCT02424617, NCT02488408, and NCT02872259). We hypothesized that Axl may be a key contributor in the establishment and growth of secondary tumors not only through its role in neoplastic cell activities but also as a promoter of proangiogenic processes. The goal of the present study was to assess the role of Axl in tumor cell induced angiogenesis and to evaluate the anti-angiogenic efficacy of the small molecule Axl-selective inhibitor, BGB324.

## RESULTS

### Axl knockdown decreases the secretion of angiogenic factors

**Figure 1:**
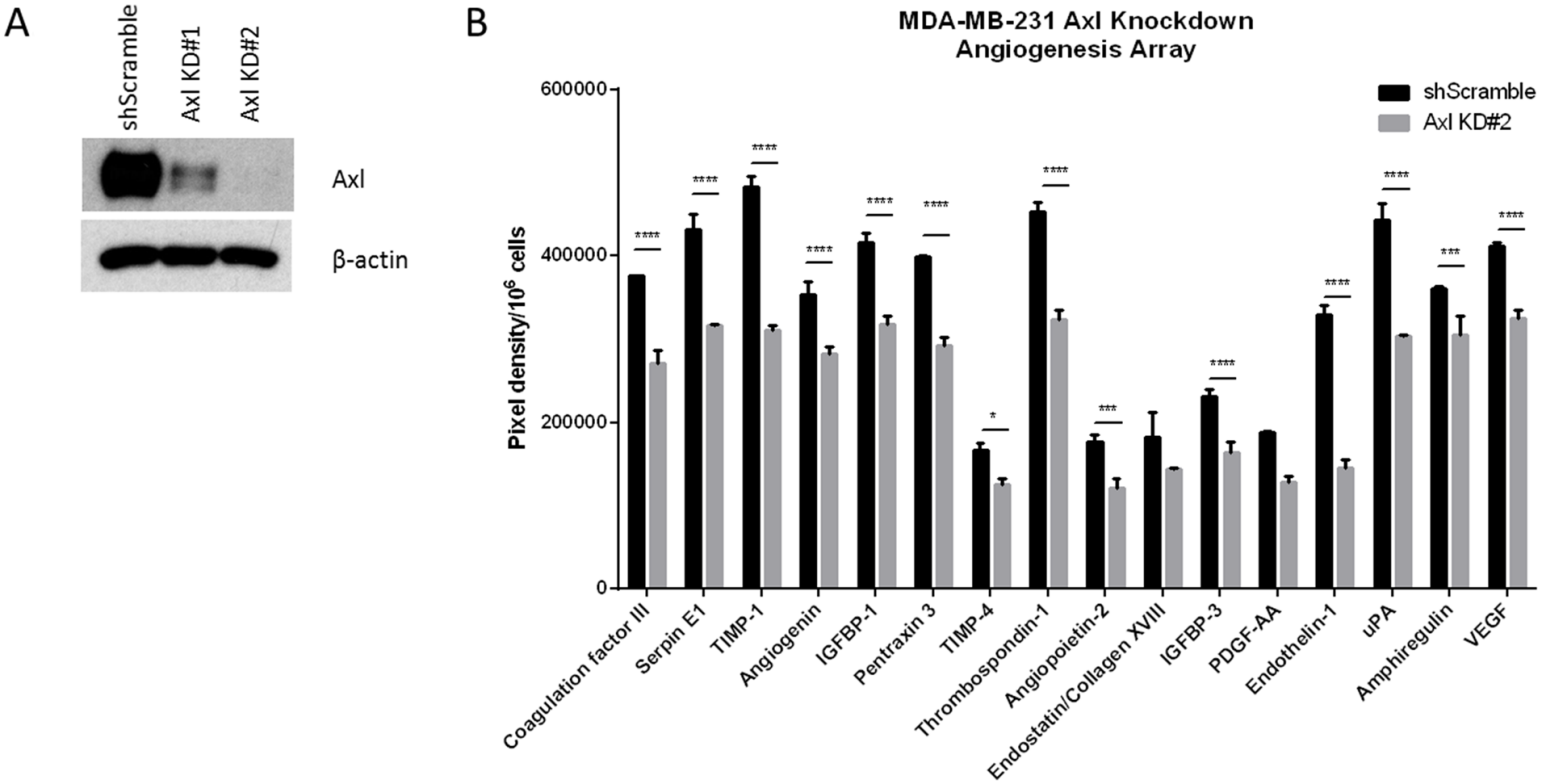
Conditioned medium from Axl knockdown breast cancer cells decreases the secretion of angiogenic factors. (**A**) Axl was genetically inactivated by shRNA in human breast cancer cell line (MDA-MB-231) and two clones were selected by Western blot for further studies. (**B**) Conditioned media of shScramble or Axl knockdown MDA-MB-231 cells were collected after 24 h and analyzed by angiogenesis array. Results are the mean and standard error values of three (*n* = 3) independent experiments. ^*^p < 0.05, ^***^p < 0.0001, ^****^p < 0.00001.

Tumor cells secrete a variety of factors that activate and modulate blood vessel formation. To determine whether Axl mediates tumor cell-induced angiogenesis, human breast cancer cells (MDA-MB-231) were transduced with lentiviral shRNA against scrambled sequence (shScramble) or Axl (shAxl) to generate stable cell lines and reductions in the level of the Axl protein in knockdown cells was confirmed by immunoblot (Figure 1A). Media collected from shScramble or shAxl cells after a 24 h exposure were analyzed for angiogenic factors using an angiogenesis array. The results showed significantly reduced levels of pro-angiogenic factors, including Thrombospondin-1, endothelin-1, uPA and VEGF, in Axl knockdown MDA-MB-231 cells compared to those detected in the shScramble control MDA-MB-231 cells (Figure 1B).

### Conditioned medium from Axl knockdown tumor cells impairs endothelial cell function *in vitro*

**Figure 2:**
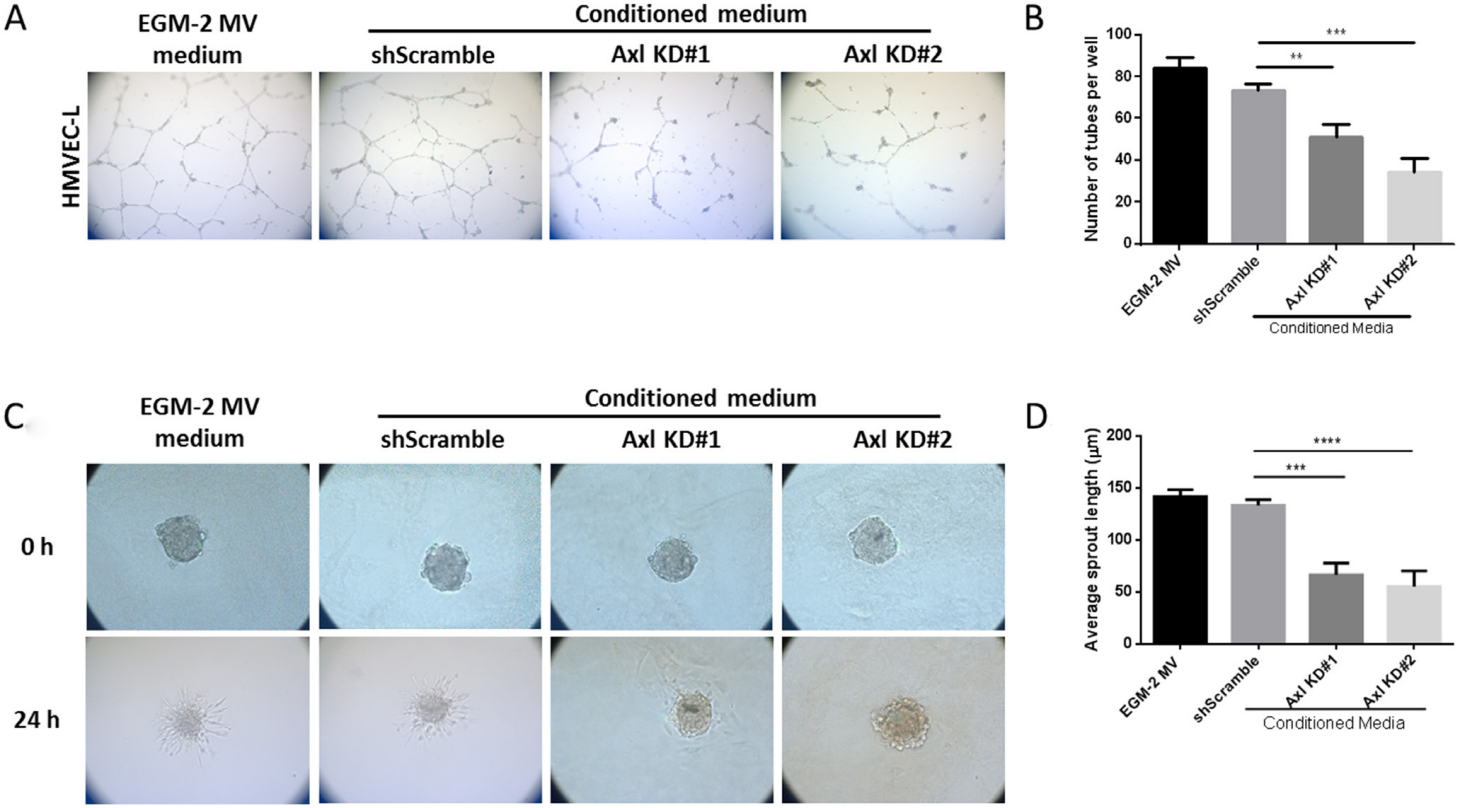
Conditioned media from Axl knockdown tumor cells impairs endothelial cell tube formation and sprouting. HMVEC cells were seeded on solidified matrigel, in the presence of tumor conditioned media or the control EGM-2MV medium. (**A**) Representative images of endothelial cell tubes formed in shScramble or Axl knockdown conditioned medium were captured by a phase contrast inverted microscope, 5X magnification. (**B**) The total number of tubes formed was quantified. HMVEC cells were suspended in 20% methylcellulose solution for 24 h to allow for spheroid formation. After 24 h, spheroids were seeded on solidified collagen, in the presence of tumor conditioned media or the control EGM-2MV medium. (**C**) Representative images of endothelial cell sprouts grown in shScramble or Axl knockdown conditioned medium were captured by a phase contrast inverted microscope, 10X magnification. (**D**) The average endothelial sprout length was measured by ImageJ. Results are the mean and standard error values of three (*n* = 3) independent experiments. ^**^p < 0.001, ^***^p < 0.0001, ^****^p < 0.00001; by two-way ANOVA.

To assess the consequence of reduced pro-angiogenic factor secretion in Axl knockdown cells on endothelial cell function, conditioned medium from Axl knockdown cells was collected and used for endothelial tube formation and sprouting assays. Formation of an extensive capillary network is facilitated by vascular cell elongation and connection with neighboring cells. Human Microvascular Endothelial Cells (HMVEC) grown in Axl knockdown tumor cell conditioned medium formed fewer endothelial tubes than HMVEC grown in the EGM2-MV medium or the medium collected from the shScramble control MDA-MB-231 cells (Figure 2A and 2B). Endothelial cell sprouting is a critical step in the induction of angiogenesis to interact with neighboring sprouts and to form a capillary network. Endothelial spheres cultured in Axl knockdown tumor cell conditioned medium had shorter sprout lengths than endothelial cells grown in the EGM2-MV medium or the shScramble control MDA-MB-231 cell conditioned medium (Figure 2C and 2D).

**Figure 3:**
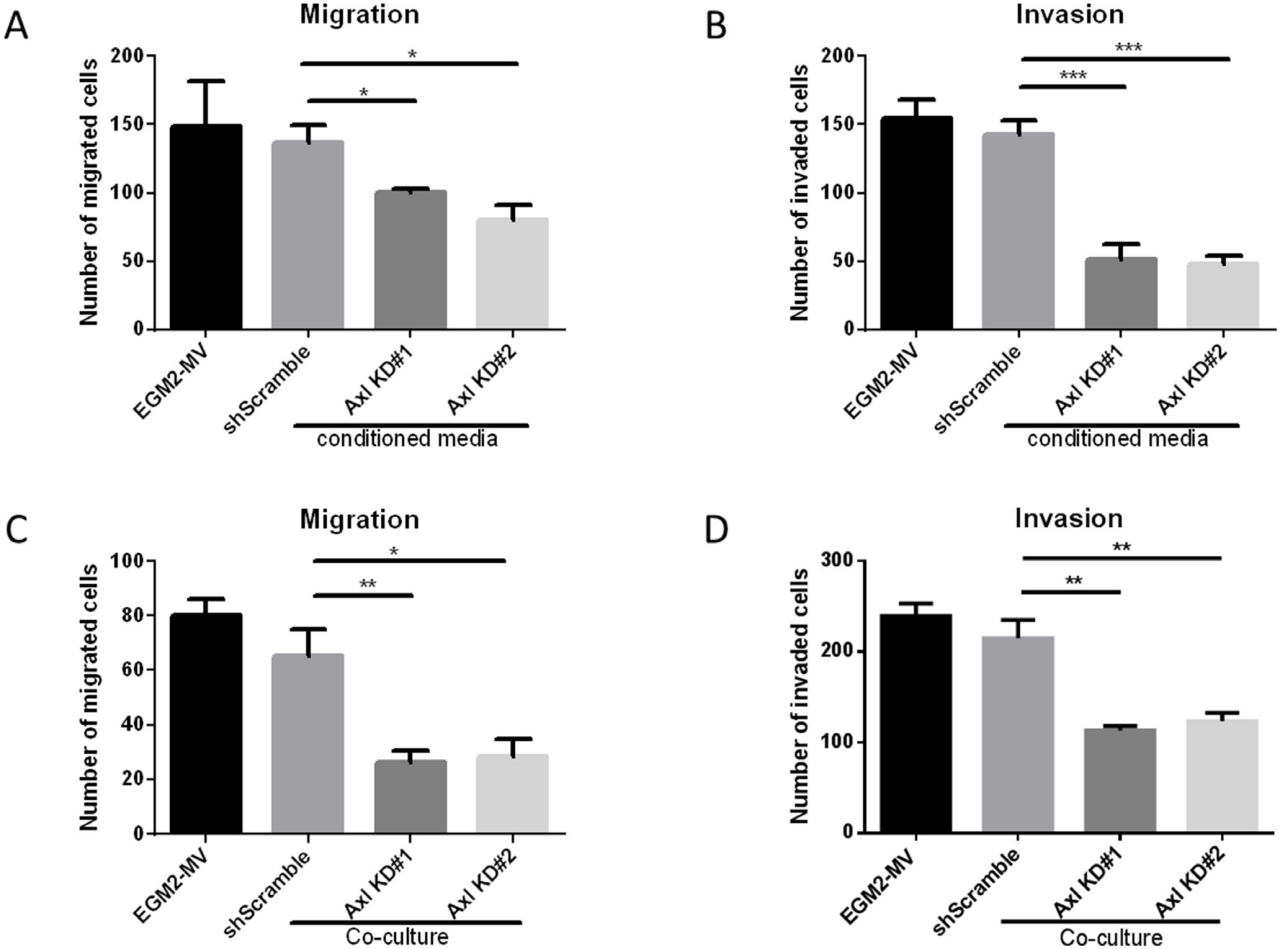
Axl knockdown of tumor cells decreases endothelial cell migration and invasion. Endothelial cells were seeded in the transwell migration or invasion chambers in the presence of EGM-2-MV medium or the tumor cell conditioned medium. The number of migrated (**A**) or invaded (**B**) endothelial cells were counted 24 h later. Tumor cells and the endothelial cells were co-cultured. Tumor cells were seeded on the bottom of the transwell chambers, and endothelial cells were seeded on the transwell chamber insert. Endothelial cells were allowed to migrate or invade for 24 h, and the number of migrated (**C**) or invaded (**D**) endothelial cells were counted. Results are the mean and standard error values of three (*n* = 3) independent experiments. ^*^p < 0.05, ^**^p < 0.01, ^***^p < 0.001; by two-way ANOVA.

To assess whether Axl knockdown tumor cell conditioned medium affects endothelial cell migration and invasion, endothelial cells were seeded in transwell migration or invasion chambers in the presence of EGM2-MV medium or the tumor cell conditioned medium. Endothelial cells showed decreased migratory and invasive capacities in the presence of Axl knockdown tumor cell conditioned medium compared to the EGM2-MV medium or the shScramble control cell conditioned medium (Figure 3A and 3B).

### Co-culture of endothelial cells and Axl knockdown tumor cells reduces endothelial cell motility and invasiveness

Since Axl knockdown tumor cell conditioned medium inhibited the angiogenic phenotypes, we evaluated whether co-culturing endothelial cells with tumor cells would demonstrate similar reductions in the migratory and invasive phenotypes. Tumor cells were seeded on the bottom of the transwell chambers and endothelial cells were seeded on the transwell chamber insert. Endothelial cells then were allowed to migrate or invade, respectively, for 24 hours. Co-culture of endothelial cells with Axl knockdown MDA-MB-231 cells showed a reduction in the ability of endothelial cells to migrate and invade compared to co-culture with shScramble control MDA-MB-231 cells or cell-free EGM2-MV medium (Figure 3C and 3D).

### Axl knockdown of tumor cells suppress tumor cell-induced angiogenesis *in vivo*

**Figure 4:**
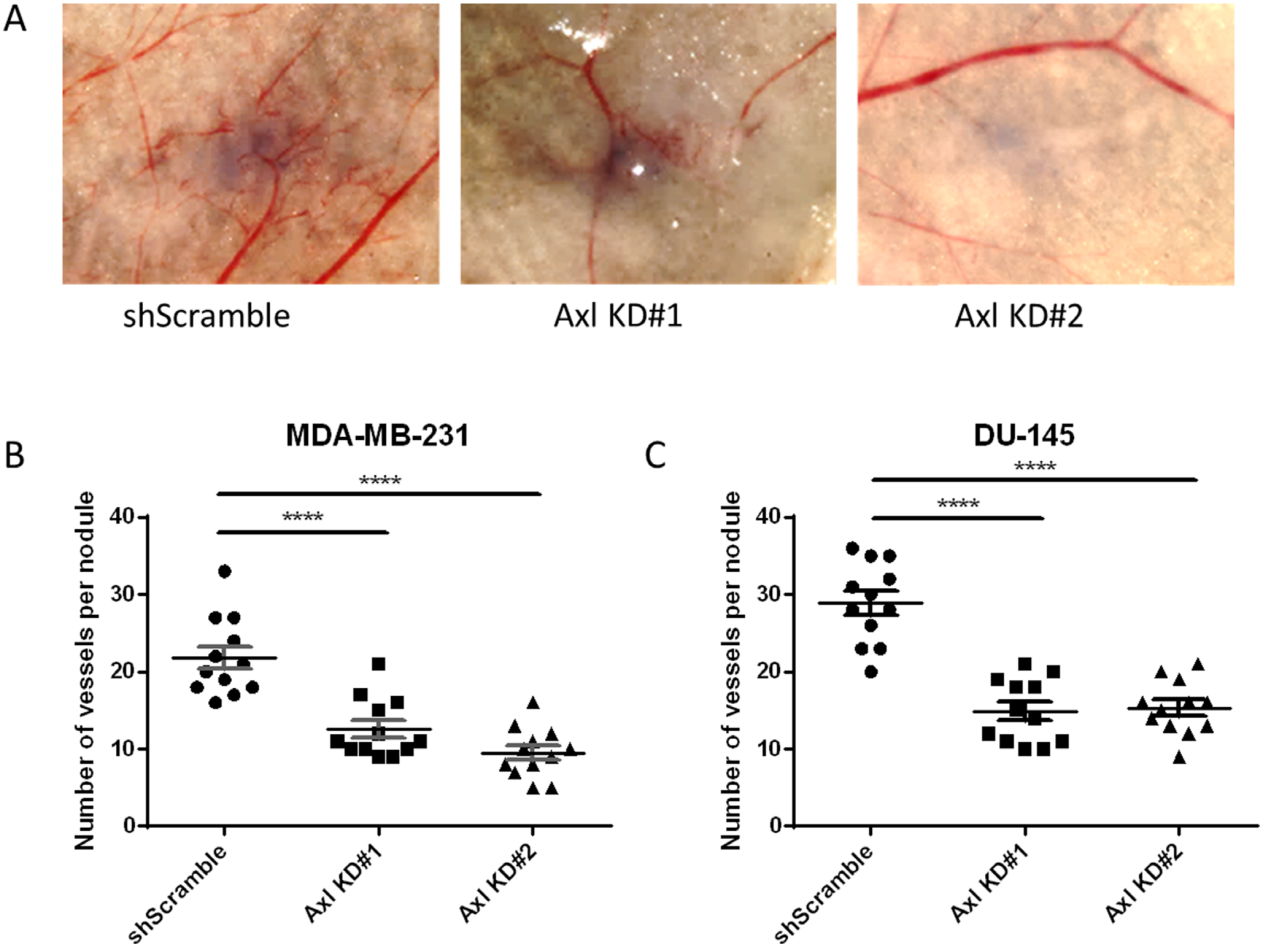
Axl knockdown of tumor cells suppresses tumor cell-induced angiogenesis *in vivo*. shScramble or Axl knockdown tumor cells were inoculated intradermally at four sites on the ventral surface of athymic nu/nu mice (10^5^ cells per nodule). (**A**) Representative images of MDA-MB-231 tumor nodules. The number of vessels recruited per tumor nodule was counted 3 days later for MDA-MB-231 (**B**) and DU-145 (**C**) cells. Results are the mean and standard error values, with twelve nodules per group analyzed. ^****^p < 0.00001; by one-way ANOVA.

To evaluate whether Axl plays a role in tumor cell-induced angiogenesis *in vivo*, Axl knockdown MDA-MB-231 breast cancer and Axl knockdown DU-145 prostate cancer cells were inoculated intradermally into the ventral skin flaps of female and male nude mice, respectively. Compared to shScramble tumor cells, both breast and prostate cancer Axl knockdown cells showed a significant decrease in the induction of tumor cell-induced angiogenesis (Figure 4A, 4B and 4C).

### BGB324 inhibits endothelial tube formation and angiogenesis *in vivo*

**Figure 5:**
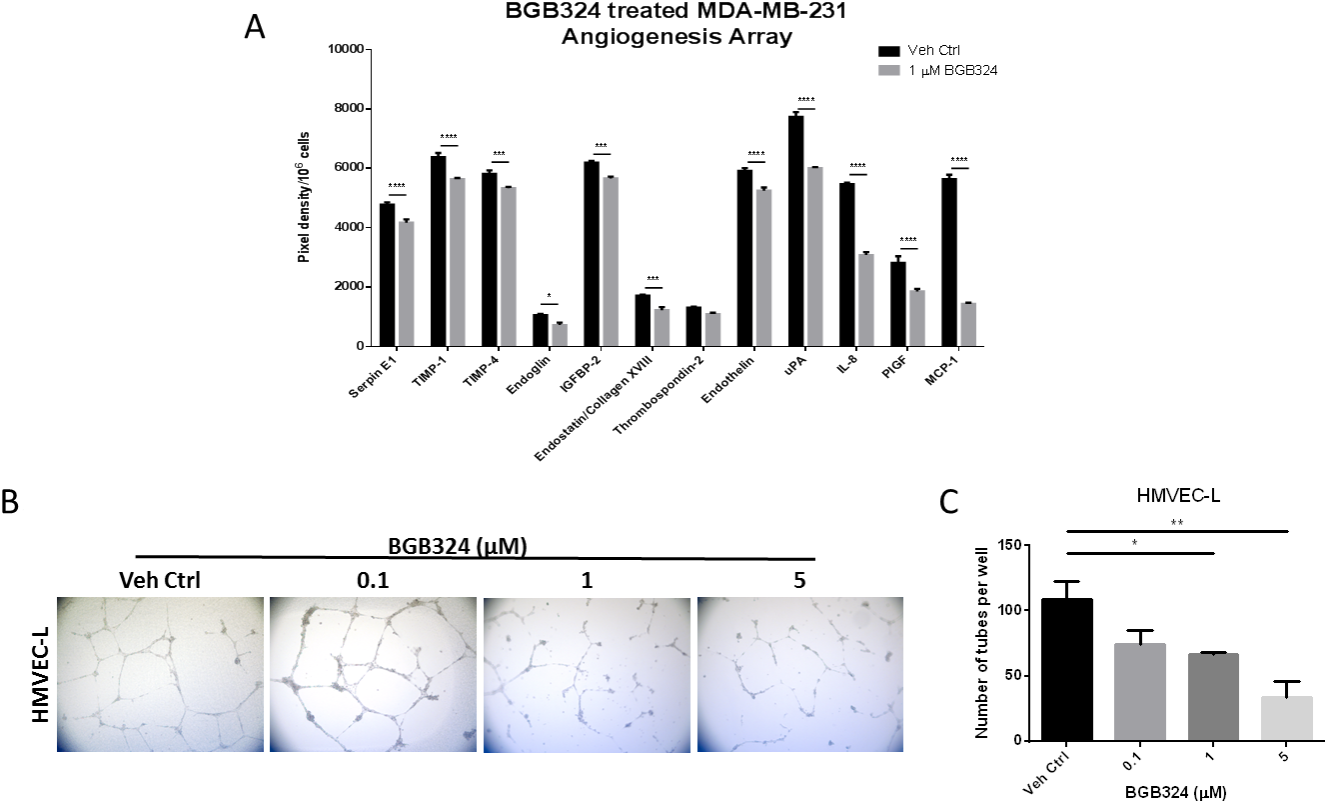
A selective Axl inhibitor, BGB324, suppresses angiogenic phenotypes. (**A**) Conditioned media of MDA-MB-231 cells treated with vehicle control (Veh Ctrl) or 1 μM BGB324 were collected after 24 h and analyzed by angiogenesis array. ^*^p < 0.05, ^***^p < 0.001, ^****^p < 0.00001. HMVEC cells were seeded on solidified matrigel in the presence of BGB324. (**B**) Representative images of endothelial cell tubes formed in the presence of BGB324, 5X magnification. (**C**) The total number of tubes formed was quantified. Results are the mean and standard error values of three (*n* = 3) independent experiments. ^*^p < 0.05, ^**^p < 0.01; by one-way ANOVA.

To assess whether pharmacologic inhibition of Axl by BGB324 would decrease the secretion of pro-angiogenic factors from tumor cells, conditioned media of tumor cells treated with drug vehicle (DMSO) or 1 μM BGB324 were collected and analyzed. Axl signaling inhibition by BGB324 decreased the secretion of pro-angiogenic factors including Endothelin, uPA, IL-8 and MCP-1 in MDA-MB-231 cells compared to vehicle treatment (Figure 5A). Furthermore, the pharmacologic inhibition of Axl by BGB324 significantly impaired the capacity of endothelial cells to form tubes in a dose-dependent manner (Figure 5B and 5C).

**Figure 6:**
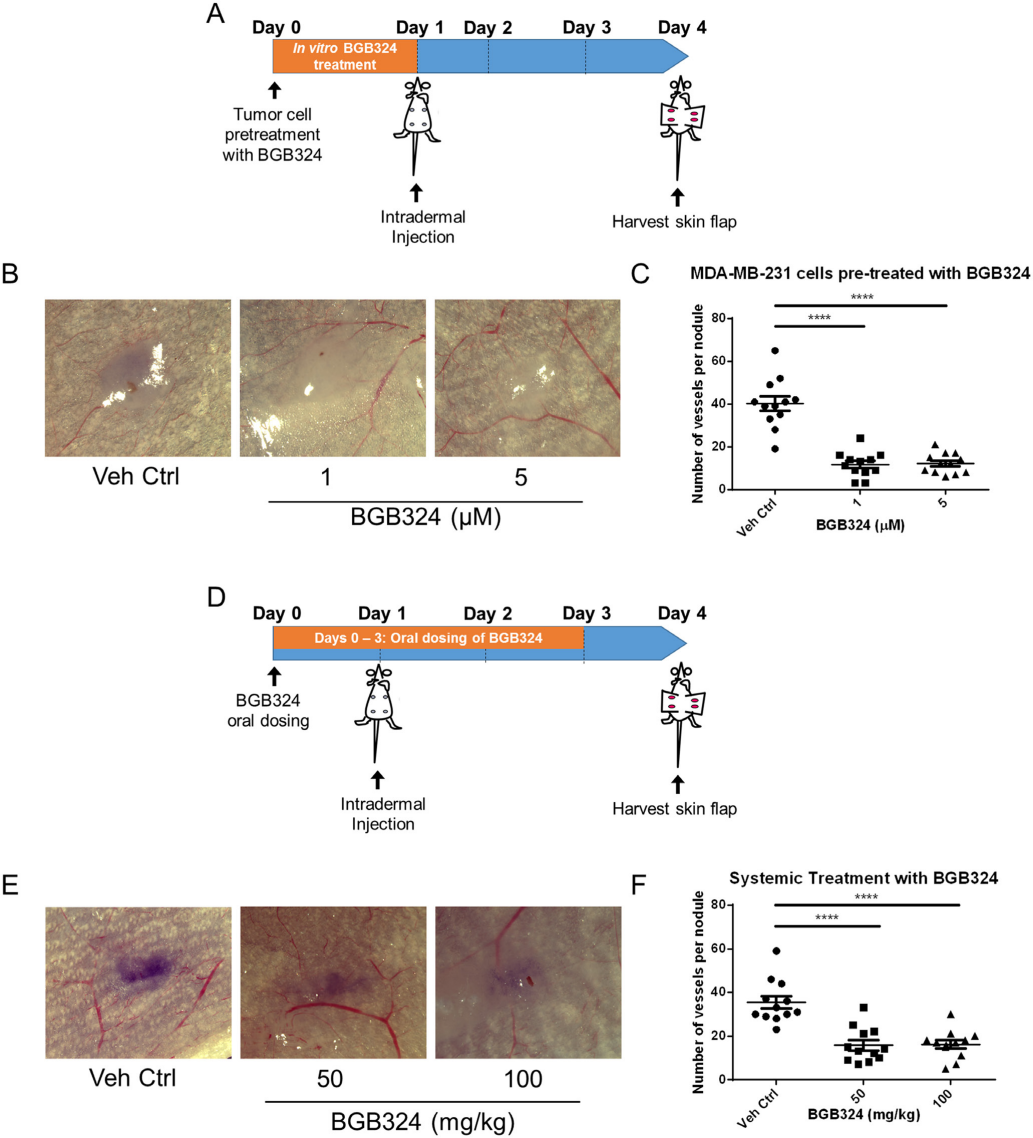
BGB324 impairs *in situ* angiogenesis. (**A**) Scheme of the intradermal assay. MDA-MB-231 cells were pre-treated with BGB324 for 24 h prior to inoculation. Tumor cells were injected intradermally at four sites on the ventral surface of athymic nu/nu mice (10^5^ cells per nodule). (**B**) Representative images of MDA-MB-231 tumor nodules where tumor cells were pre-treated with BGB324 for 24 h prior to inoculation. (**C**) The number of vessels recruited per tumor nodule was counted three days later for mice inoculated with pre-treated MDA-MB-231 cells: vehicle control (Veh Ctrl), 1-, or 5- μM BGB324 for 24 h prior to tumor cell inoculation. (**D**) Scheme of the intradermal assay. Daily oral dosing with BGB324 (50- or 100- mg/kg BGB324) or agent vehicle control began a day before tumor inoculation and continued for 3 days. MDA-MB-231 cells were inoculated intradermally at four sites on the ventral surface of athymic nu/nu mice (10^5^ cells per nodule). (**E**) Representative images of MDA-MB-231 tumor nodules with systemic BGB324 treatment. (**F**) The number of vessels recruited per tumor nodule was counted three days later for mice inoculated with MDA-MB-231 cells. Mice received vehicle control (Veh Ctrl), 50-, or 100- mg/kg BGB324 via oral gavage. Results are the mean and standard error values, with twelve nodules per group analyzed. ^****^p < 0.00001; by one-way ANOVA.

Since pharmacologic Axl inhibition decreased the secretion of angiogenic factors, we also assessed the effect of pharmacologic Axl inhibition in tumor cells on their *in vivo* angiogenic potential. Tumor cells were treated with BGB324 for 24 h prior to inoculating them intradermally into the ventral skin flaps of female nude mice (Figure 6A). Compared to vehicle treated MDA-MB-231 cells, BGB324-treated MDA-MB-231 cells showed a significant decrease in their angiogenic capacity (Figure 6B and 6C). To determine whether systemic treatment of mice with BGB324 could similarly result in a reduction in tumor cell induced angiogenesis, female nude mice were gavaged with either vehicle control, 50 mg/kg or 100 mg/kg of BGB324 once daily for three days (Figure 6D). The results demonstrated that 50 and 100 mg/kg BGB324 treatment significantly decreased the ability of MDA-MB-231 tumor cells to initiate a vascular network (Figure 6E and 6F).

## DISCUSSION

The growth of primary tumors and metastatic neoplastic lesions is strongly dependent on the cancer cells’ ability to initiate their own blood supply. Hence, angiogenesis, the formation of new blood vessels from pre-existing vasculature, is an important hallmark of tumor progression and metastasis [1, 2] and a key anti-cancer target [41].

Tumor cells secrete a variety of factors that activate and modulate blood vessel formation. Upregulation of receptor tyrosine kinases in tumor cells promote expression and secretion of these pro-angiogenic factors [17, 18], as well as other metastatic phenotypes including tumor cell migration, invasion, proliferation and survival [42]. Many molecular signaling pathways mediate functional components of tumor angiogenesis including VEGF/Neuropilin signaling in endothelial cell migration, Notch signaling in endothelial cell sprouting, and Angiopoietin/Tie2 signaling in endothelial cell proliferation and enhancement of tight junctions [43].

Axl, a pro-tumorigenic receptor tyrosine kinase, is expressed ectopically on tumor cells, but also on host stromal cells, including endothelial cells [33, 34]. Axl is required for VEGF-A dependent angiogenesis [44, 45] and that VEGF-A dependent activation of VEGFR-2 transactivates Axl to promote downstream signaling pathways [45]. Although the present study did not identify downstream signaling pathways of Axl mediating angiogenic functions, Maacha *et al.* observed that Axl mediates lysosome trafficking and secretion of the proteolytic enzyme cathepsin B [46]. Our present finding shows that Axl suppression of tumor cells by knockdown or BGB324 contributes to decreased secretion of factors known to promote angiogenesis [47–53], including thrombospondin-1, endothelin-1, uPA and VEGF (Figures 1B and 5A). Therefore, our findings suggest that Axl signaling promotes angiogenesis through secretion of various pro-angiogenic factors. Indeed, Chen *et al.* demonstrated that BGB324 contributes to the accumulation of autophagosomes and lysosomes, and consequently, promotes apoptosis [54]. Our results and previous studies by others show that Axl inhibition decreases expression and secretion of proteolytic enzymes including uPA, matrix metalloproteases and cathepsins [46, 55]. These proteolytic enzymes are known to promote steps within the metastatic cascade, including the facilitation of tumor cell migration and invasion, as well as endothelial cell tube formation and sprouting [14, 56–58]. Our assessment of the endothelial cell functions using the tumor cell conditioned medium suggest that Axl promotes tumor cell-induced angiogenic processes, including endothelial cell tube formation, sprouting, migration and invasion, and *in situ* angiogenesis.

Since Axl is known to mediate many pro-tumorigenic and metastatic functions including cell migration, invasion, proliferation and survival in a variety of tumor models [30, 32, 59], there has been a considerable interest in targeting the Axl signaling pathway. BGB324 is a highly selective Axl inhibitor that is currently in phase I and II clinical trials. Pharmacologic Axl inhibition in the human umbilical vein endothelial cells decreased *in vitro* functions associated with angiogenesis, such as endothelial tube formation [35]. Consistent with phenotypes observed using the Axl knockdown tumor cell lines, our results suggest that BGB324 impairs endothelial cell tube formation (Figure 5B and 5C). In addition, BGB324 decreased the *in situ* induction of angiogenesis by tumor cells treated with BGB324 for 24 h prior to tumor cell inoculation, as well as tumor bearing mice treated systemically with BGB324 (Figure 6). Lei *et al.* and Kanlikilicer *et al.* demonstrated that Axl inhibition decreases immunohistochemical staining of the endothelial cell marker CD31 [60, 61]. Our results suggest that such decrease in CD31 staining can result from the inhibition of Axl signaling in tumor cells as well as through direct inhibition of endothelial cell Axl signaling.

In summary, the present results demonstrate that Axl is a novel anti-angiogenic target in both tumor and endothelial cells. Inhibition of Axl in tumor cells reduced secretion of pro-angiogenic factors and indirectly suppressed the recruitment of endothelial cells to the tumor mass. In endothelial cells, Axl targeting directly inhibited functions associated with angiogenesis. Collectively, our findings indicate that Axl promotes tumor cell induced angiogenesis and thus is a promising therapeutic target to impair tumor progression and metastasis.

## MATERIALS AND METHODS

### Cell culture

Human lung microvascular endothelial cells (HMVEC-L) was obtained from Lonza, and human breast and prostate cancer cell lines (MDA-MB-231 and DU-145, respectively) were obtained from ATCC. HMVEC-L cells were cultured in EGM2-MV media supplied by Lonza (CC-3202). The human breast cancer cell line MDA-MB-231 and human prostate cancer cell line DU-145 were cultured in DMEM and Eagle’s essential medium supplemented with 10% fetal bovine serum, 1% L-glutamine and 1% penicillin-streptomycin. All cells were maintained at 37°C in a humidified atmosphere of 5% CO_2_. Mycoplasma tests were performed in house by using MycoAlert Mycoplasma Detection Kit (Lonza, Walkersville, MD, USA).

### Reagents

BGB324 was obtained from Selleckchem (Houston, TX, USA). For *in vitro* evaluations, BGB324 was dissolved in sterile DMSO and stored at –20°C. For *in vivo* assays, BGB324 was formulated in 0.5% hydropxypropylmethylcellulose + 0.1% Tween 80.

### Generation of stable Axl knockdown cell line using shRNA

Axl knockdown MDA-MB-231 and DU-145 cell lines were generated with Mission Lentivirus Transduction particles (Sigma-Aldrich, St. Louis, MO, USA). Scrambled non-silencing shRNA (SHC202V) or Axl shRNA (MDA-MB-231 Axl KD#1: TRCN0000001039, Axl KD#2: TRCN0000001040; DU-145 Axl KD#1: TRCN0000001040, KD#2: TRCN0000001041) was transduced in MDA-MB-231 and DU-145 cells. When cells reached 50–60% confluence in a 6-well plate, cells were infected with 6 μg/mL polybrene (Millipore, Burlington, MA, USA) and lentivirus. After 48 h, the medium was changed and the cells were selected with 3.6 μg/mL of puromycin (Thermo Fisher Scientific, Waltham, MA, USA). Knockdown cells were maintained under puromycin selection for the duration of the experiments.

### Collection of Axl-KD cell conditioned medium

For experiments using conditioned media, cells were seeded (5 × 10^4^ cells per well) in a 24-well plate. After 24 h, the medium was changed to EGM2-MV (0.5 ml per well). The tumor cell conditioned medium was collected and centrifuged at 300 × *g* for 10 min to remove cellular debris. The supernatant subsequently was used for experiments.

### Angiogenesis microarray

Scrambled non-silencing shRNA control or Axl knockdown MDA-MB-231 cells were seeded in a 60-mm dish. When the cells reached 50–60% confluence, the medium was replaced with serum free medium. Cell conditioned media were harvested 24 h later, centrifuged at 300 *×* *g* for 10 min to remove cell debris. Cells from each sample were trypsinized and counted for normalization of secreted levels with total cell number. The angiogenic factors secreted in the conditioned media were measured using the human angiogenesis array (R&D Systems, Minneapolis, MN, USA), according to the manufacturer’s instructions. Briefly, the blot was incubated with conditioned media. After washing and incubating the blot with HRP-conjugated secondary antibody, ChemiReagent Mix was used for chemiluminescent detection on x-ray film. The pixel density was normalized to 10^6^ cells.

### Tube formation assay

Formation of an extensive capillary network *in vitro* was measured in a tube formation assay as described previously [62]. Each well of the 24 well plate was rinsed with PBS, then 200 μL matrigel (Corning) was added and solidified at 37°C for 30 min. 2.5 × 10^4^ HMVEC-L cells were seeded on solidified matrigel, in the presence of tumor conditioned media or BGB324, and incubated at 37°C for 24 h. Endothelial tubes were quantified and imaged using Leica DM4000 B LED microscope.

### Methylcellulose solution

To prepare methylcellulose solution, 1.2 g of methylcellulose was autoclaved in a 250 mL Erlenmeyer flask. EGM2-MV medium was pre-warmed to 60°C in a water bath, and 50 mL of pre-warmed medium was added to the autoclaved methylcellulose. The methylcellulose solution was stirred at room temperature for 20 min. Additional 50 mL of room temperature EGM2-MV was added and stirred at 4°C for 2 h. The final solution was centrifuged at 5,000 × *g* for 2 h at room temperature to remove undissolved methylcellulose. The supernatant was used for the sprouting assay.

### Sprouting assay

HMVEC-L cells (7.5 × 10^4^ cells) were suspended in 15 mL of EGM-2 medium containing 20% methylcellulose solution. 150 μL of the cell mixture was added to each well (750 cells per well) of a 96 U-shaped well suspension plate (Cellstar), and incubated at 37°C for 24 hours to allow for spheroid formation. After 24 h, spheroids were collected using a 5 mL serological pipette and centrifuged at 300 × *g* for 10 minutes. Rat tail collagen type-I was diluted to 2 mg/mL in EGM2-MV and neutralized to pH 7.0 by drop-wise addition of 1 N NaOH. Each well of the 12 well plate was rinsed with PBS, then 100 μL collagen was added and solidified at 37°C for 30 min. The spheroids were resuspended into 1.4 mL collagen solution, and 100 μL of spheroid suspension was added to the top of solidified collagen gel. The second layer of spheroid-collagen mixture was allowed to set for 1 h. EGM2-MV or tumor conditioned medium were added to each well and incubated at 37°C for 24 hours. Endothelial cell sprouting was quantified and imaged using Leica DM4000 B LED microscope.

### Transwell chamber assays

HMVEC-L cell migration was examined using a transwell insert with 8 μm pores membrane (BD, Franklin Lakes, NJ, USA). 1 × 10^3^ HMVEC-L cells were seeded in the inserts with complete medium or tumor conditioned medium, allowed to migrate for 24 h, after which, the cells on the underside of the insert were stained with crystal violet and counted. In the invasion assay, inserts were coated with Matrigel. 5 × 10^3^ HMVEC-L cells were suspended in serum-free media and loaded into the insert. Complete medium or the tumor cell conditioned medium was used in the lower chamber as a chemo-attractant. After 24 h, the cells on the underside of the insert were stained and counted. In a co-culture system, 5 × 10^4^ MDA-MB-231 cells were seeded in a 24 well plate and were allowed to adhere for 48 h. Media was changed to EGM-2 and appropriate number of HMVEC-L cells (migration: 1 × 10^3^ cells; invasion: 5 × 10^3^ cells) were seeded in the transwell. After 24 h, the cells on the underside of the insert were stained and counted.

### Intradermal assay

Induction of angiogenesis was measured in an intradermal assay as described previously [14, 63]. Scramble non-silencing shRNA control or Axl knockdown cells, or tumor cells pre-treated with BGB324 prior to inoculation (1 × 10^5^ cells/10 μl) were injected intradermally at four sites on the ventral surface of four- to six- week old athymic nu/nu mice. Prior to tumor cell inoculation, one drop of 0.4% trypan blue stain was added to the cell suspension for tumor nodule visualization *in vivo*. Three days later, the mice were euthanized and their skin flaps were removed and analyzed. Tumor angiogenesis was evaluated by counting the number of blood vessels growing into the tumor nodule using a Leica M216F stereomicroscope. Tumor nodule images were captured using a Retiga EXi Fast 1394 camera and Volocity software. For systemic treatment of mice, oral dosing with BGB324 (50 mg/kg or 100 mg/kg BGB324) or vehicle control once daily began a day before tumor inoculation and continued to day 2. All *in vivo* experiments were approved by the University of Florida Institutional Animal Care and Use Committee.

### Statistical analysis

Data are expressed as means ± SEM. Student’s *t* test was applied unless otherwise noted. All statistical analysis was performed using GraphPad Prism 5.0 software (San Diego, CA, USA). A threshold of *P* < 0.05 was designed as statistically significant.

## Abbreviations

IGFBP-1: insulin-like growth factor-binding protein 1
IGFBP-2: insulin-like growth factor-binding protein 2
IGFBP-3: insulin-like growth factor-binding protein 3
IL-8: interleukin-8
MCP-1: monocyte chemoattractant protein 1
PIGF: phosphatidylinositol-glycan biosynthesis class F
PDGF-AA: platelet-derived growth factor AA
TIMP-1: tissue inhibitor of metalloproteinase 1
TIMP-4: tissue inhibitor of metalloproteinase 4
uPA: urokinase-type plasminogen activator
VEGF: vascular endothelial growth factor
